# Factors associated with adherence to BRCA1/2 mutation testing after oncogenetic counseling in long-surviving patients with a previous diagnosis of breast or ovarian cancer

**DOI:** 10.1007/s12687-023-00671-x

**Published:** 2023-09-19

**Authors:** Silvia Actis, Marta D’Alonzo, Luca Pace, Serena Mucciacito, Valentina Elisabetta Bounous, Luca Giuseppe Sgrò, Matteo Mancarella, Annamaria Ferrero, Nicoletta Biglia

**Affiliations:** 1 Gynecology and Obstetrics Unit, Mauriziano Umberto I Hospital, Department of Surgical Sciences, University of Turin, Largo Filippo Turati, 62, 10128 Turin, Italy; 2 Gynecology and Obstetrics Unit, Mauriziano Umberto I Hospital, 10128 Turin, Italy

**Keywords:** BRCA1/2, Oncogenetic counseling, Carrier testing, Decision making

## Abstract

BRCA1/2 mutations account for 5 to 10% of breast and 15% of ovarian cancers. Various guidelines on BRCA1/2 genetic counseling and testing have been issued, and the criteria have evolved over the years. Oncogenetic counseling aims to inform patients about the possibility and implications of undergoing predictive testing and risk management programs. We analyzed a cohort of 50 subjects with a previous personal history of breast or ovarian cancer who had not been tested for BRCA1/2 mutations at the time of diagnosis but were found eligible according to the most recent guidelines. All patients were offered pre-test oncogenetic counseling and BRCA1/2 genetic testing. The mean time from cancer diagnosis to genetic counseling was over 10 years. We analyzed socio-demographic and psychological parameters associated with the decision to undergo BRCA1/2 genetic testing or the reasons behind the withdrawal. Thirty-nine patients underwent BRCA1/2 genetic testing. Patients who accept the genetic test communicate more easily with family members than those who refuse. Factors associated with test refusal are having a long-term partner and having a negative perception of life. There is a trend, although not statistically significant, toward younger age at cancer diagnosis, more likely to participate in cancer screening programs (71.8% vs. 45.5%), and more likely to have daughters (63.3% vs. 37.5%) in the group that accepted the test. The offer of BRCA testing was well accepted by our study population, despite the many years since the cancer diagnosis. With the perspective of further broadening the access criteria to genetic testing, it is important to understand how to best approach pre-test counseling in long-surviving patients with a previous diagnosis of cancer.

**Supplementary Information:**

The online version contains supplementary material available at 10.1007/s12687-023-00671-x.

## Introduction

Advances in the field of human genetics have led to the development of genetic tests now commonly used in clinical practice (Collins et al. 2001, Laberge and Burke 2008, Guttmacher et al. 2010). Among these analyses, those related to the BRCA1 and BRCA2 genes emerge as extremely important since their mutations are responsible for approximately 5 to 10% of breast and 15% of ovarian cancers (Melchor and Benítez 2013). The genetic testing offering represents an opportunity for high-risk patients to know their carrier status and thus their susceptibility to breast and ovarian cancer. Criteria for access to the test have expanded over the years, allowing more individuals to access genetic testing. The management of patients carrying BRCA1/2 mutations includes adherence to surveillance, chemoprophylaxis, and/or prophylactic surgery pathways (Clinical Practice Guidelines and in Oncology (NCCN Guidelines®) Genetic, familial high-risk assessment: breast, ovarian, and pancreatic 2022; Paluch-Shimon et al. 2016). The knowledge of the mutation status is of great importance not only for the person who has already developed the cancer but also for the family members. Testing eligible individuals, even several years after cancer diagnosis and in older individuals, is an opportunity to identify a mutation in a family. Knowledge of a BRCA1/2 mutation in healthy patients may allow prophylactic measures to be taken that can reduce the incidence of breast and ovarian cancer and reduce mortality in this population (Nelson et al. 2019). However, it should not be overlooked that the knowledge of the BRCA1/2 test result has psychological implications (Lombardi et al. 2019).

The clarity of pre-test counseling, in which all the tools for understanding the results must be provided, is crucial to prevent the decision to undergo genetic testing from being made unknowingly. Oncogenetic counseling is a complex and evolving field that requires continuous updating of practitioners and readjustment of clinical practice to the needs of the consultees (Lerman et al. 1997, Lerman et al. 1995, Shatz et al. 2015).

The information strategy used in oncogenetic counseling, based on “educating the subject,” has been compared in some studies with other pre-test information methods. It has been found to facilitate more in-depth processing of the information provided during the session, thus improving the individual’s ability to make conscious decisions (Lerman et al. 1997, 1995). Previous studies have investigated the impact of pre-test counseling and the possibility of knowing one’s mutational status at the time of cancer diagnosis (Meijers-Heijboer et al. 2003; Ardern-Jones et al. 2005). In our study, we investigated how socio-demographic, medical, psychological factors, risk perception, personal and/or family history of cancer, and psychological distress can impact adherence to genetic testing for BRCA1 and 2 gene mutation years after a previous cancer diagnosis.

## Methods

The study was conducted at the BRCA outpatient clinic of the Gynecology and Obstetrics Unit of the Mauriziano Umberto I Hospital in Turin. The Institutional Ethics Committee of Mauriziano Umberto I Hospital approved the study protocol (Prot. No. 83747, date of approval: 07/28/2022).

The selection of patients was performed by systematically searching all electronic medical records of patients with a personal history of breast and/or ovarian cancer who underwent surgery or follow-up visits at the SCDU Gynecology and Obstetrics of the Mauriziano Umberto I Hospital from 1989 to June 2022.

All patients eligible for genetic testing for BRCA1/2 mutations by personal cancer history according to the latest AIOM (Associazione Italiana Oncologia Medica, Italian Medical Oncology Association) guidelines (May 2021) (AIOM Recommendations for implementation of predictive and preventive BRCA testing in breast, ovarian 2021) were identified:

- male with breast cancer
- female with breast and ovarian cancer
- female with breast cancer < 36 years old
- female with triple-negative breast cancer < 60 years old
- female with bilateral breast cancer < 50 years old
- female with nonmucinous, non-borderline ovarian carcinoma at any age

All patients already tested for BRCA1/2 genetic mutation were excluded, and eligible patients were reached telephonically from July to September 2022. In this telephone contact, the concepts of BRCA1/2 susceptibility and genetic risk were introduced, and the possibility of undergoing pre-test counseling and subsequent genetic testing was communicated.

Patients who accepted pre-test genetic counseling at our institute scheduled an appointment and were properly informed about the implications of an informative genetic test result. During this visit, directions and documentation were also provided for the genetic test blood draw at the Molinette Hospital Medical Genetics Institute.

We have prepared 3 different questionnaires:

- Questionnaire A (Supplementary file 1) to collect anamnestic history, socio-demographic indicators, and medical comorbidities
- Questionnaire B (Supplementary file 2) to investigate in decision-making context and to assess the rationale behind the decision to access oncogenetic counseling. Questionnaire B is divided into two sections, the first deals with the influences of personal and/or family history of cancer, the perceived risk, and the role of family cohesion on the decision to pursue genetic testing; the second part consists of the 15 items of the Event Impact Scale (IES) (Horowitz et al. 1979). The IES is a tool that assesses the impact of a distressing experience and measures stress-related symptomatology by considering intrusion and avoidance of thoughts or feelings related to the experience. The decision to undergo oncogenetic counseling and BRCA1/2 testing was taken as a stressful event. Patients were asked to recall the emotions they felt since the initial telephone consultation and the counseling session.
- Following the pre-test counseling, patients who joined the study were re-contacted by telephone to learn about their actual adherence to BRCA1/2 genetic testing. Those who decided not to undergo the genetic test were given questionnaire C (Supplementary file 3) concerning the reasons behind their refusal and presented only to subjects who declined genetic testing after counseling. This choice was made with the goal of potentially addressing this population of individuals who declined genetic testing by better-structuring counseling in the future.

Questionnaires A and B were administered in person at the time of oncogenetic counseling and were collected by study personnel before patients left our center; these two questionnaires were addressed to all study participants. Questionnaire C was administered by telephone at the time of the telephone follow-up visit only to participants who decided not to undergo genetic testing.

The statistical software IBM SPSS (1.0.0.1213) was used for analysis. Student's *t*-test was used to analyze the difference between continuous variables in the two independent samples. Pearson’s chi-square test or Fisher’s exact test as appropriate were used to analyze the joint distribution of quantitative variables. Psychological parameters were transformed into binary variables based on cut-offs. A statistical significance level of 0.05 was set, and therefore, *p*-values < 0.05 were considered statistically significant.

## Results

One hundred thirty-one patients, 19 men, and 112 women, were considered eligible for the study. Seventy-two patients were lost at first contact, including 64 who died and 8 whose telephone numbers were no longer active and could not be traced. Fifty-nine patients were contacted by telephone: 6 refused counseling and 53 agreed to make an appointment at the BRCA outpatient clinic of the Mauriziano Umberto I Hospital. Three patients canceled the counseling visit, and only 50 patients joined the study and answered the questionnaires.

The sample consisted of 50 subjects, 49 women and 1 man, aged 43 to 87 years, with a mean age of 61 years (S.D. 1.55).

After pre-test counseling, approximately 1 month after counseling, 39 patients (78%) underwent BRCA1/2 genetic testing (test group, TG), while 11 (22%) refused (no-test group, NTG). Among the latter only 3, in the context of second telephone contact, preferred not to share the reasons for their decision and therefore did not respond to questionnaire C.

The TG consisted of 38 women and one man, while the NTG consisted of 11 women.

Of the 39 subjects who decided to undergo testing, 2 had a positive result for class 5 mutation; of these two, one patient was diagnosed with ovarian cancer at the first visit with transvaginal ultrasound. In 29 subjects, no mutation was identified, and 8 have not yet received the result.

- *Questionnaire A*

TG patients showed a trend towards a younger mean age at cancer diagnosis of 45.3 years (S.D. = 1.57) compared to the NTG with a mean age of 52.3 years (S.D. = 3.79) (*p* = 0.056); this difference appears remarkable, but not statistically significant. The mean time from cancer diagnosis to genetic testing prescription was similar between the two groups: 14.4 years (S.D. = 2.3) for the TG and 13.2 years (S.D. = 3.97) for the NTG (*p* = 0.65). The mean age at the time of the genetic testing proposal was 59.7 years (S.D. = 1.63) for the TG and 65.5 years (S.D. = 3.88) for the NTG (*p* = 0.126).

Socio-demographic indicators, medical comorbidities of the sample under study, and the personal context obtained through questionnaire A are shown in Table 1.

**Table 1:** Socio-demographic indicators, medical comorbidities of the sample under study, and the personal context of the test group and no-test group. S.D.: standard deviation

		Test-group *n*. 39 (%)	No-test group *n*. 11 (%)	*p*-value
Age at the time of cancer diagnosis (years)		45.28 (S.D. 1.57)	52.27 (S.D. 3.78)	0,056
Sex	Female	38 (97.4)	11 (100)	0.960
	Male	1 (2.6)	0 (0)	
Previous cancer diagnosis	Only breast cancer	34 (87.2)	8 (72.7)	0. 248
	Only ovarian cancer	5 (12.8)	1 (9.1)	
	Breast cancer and ovarian cancer	0 (0)	2 (18.2)	
Time since the first cancer diagnosis (years)		14.4 (S.D. 2.3)	13.2 (S.D. 3.97)	0,65
Age at the time of genetic testing proposal (years)		59.7 (S.D. 1.63)	65.5 (S.D. 3.88)	0.126
Education level	Bachelor/master/doctor degree	5 (12.8)	3 (27.3)	0.248
	Elementary/middle/high school diploma	34 (87.2)	8 (72.7)	
Occupational status	Stable employment	32 (82.1)	9 (81.8)	0.986
	unstable employment	7 (17.9)	2 (18.2)	
Marital status	Stable partner	23 (59)	10 (90.9)	0.048
	Without a partner	16 (41)	1 (9.1)	
Daughters	Yes	19 (63.3)	3 (37.5)	0.189
	No	11 (36.7)	5 (62.5)	
Sons	Yes	22 (73.3)	6 (75)	0.924
	No	8 (26.7)	2 (25)	
Comorbidity	At least one comorbidity	27 (69.2)	7 (63.6)	0.725
	No comorbidity	12 (30.8)	4 (36.4)	
Personal medical history of depression	Yes	12 (30.8)	4 (36.4)	0.725
	No	27 (69.1)	7 (63.6)	
Chronic therapy	At least one medication	29 (74.4)	8 (72.7)	0.913
	No medication	10 (25.6)	3 (27.3)	
Alcohol consumption	Yes	24 (61.5)	6 (54.5)	0.676
	No	15 (38.5)	5 (45.5)	
Smoking habit	Yes	10 (25.6)	0 (0)	0.060
	No	29 (74.4)	11 (100)	
Adherence to the vaccination campaign for SARS‑CoV‑2	Yes	36 (92.3)	11 (100%)	0.343
	No	3 (7.7)	0 (0)	
Adherence to screening campaigns for breast and/or cervical cancer	Yes	28 (71.8)	5 (45.5)	0.103
	No	11 (28.2)	6 (54.5)	

Subjects in the NTG were more likely to have a partner (*p* = 0.048). Subjects in the NTG show a lower tendency to tobacco use than the TG (*p* = 0.06), and adherence to screening campaigns for breast and/or cervical cancer tends to be higher in the TG (71.8%) compared to the NTG (45.5%) (*p* = 0.103); these differences appear noteworthy, although not significant.

A higher proportion of patients in the TG has daughters, 63.3% vs 37.5% in the NTG (*p* = 0.189), although these results do not reach statistical significance, while the percentage of male offspring looks superimposable.

In both samples, perceived knowledge about BRCA1/2 gene mutations was overlapping, in the TG, 23% considered themselves well-informed, 29% had heard of the topic, and 28% had never heard of it, compared to 27%, 27%, and 46% of the NTG, respectively.

- *Questionnaire B*

**Fig. 1:**
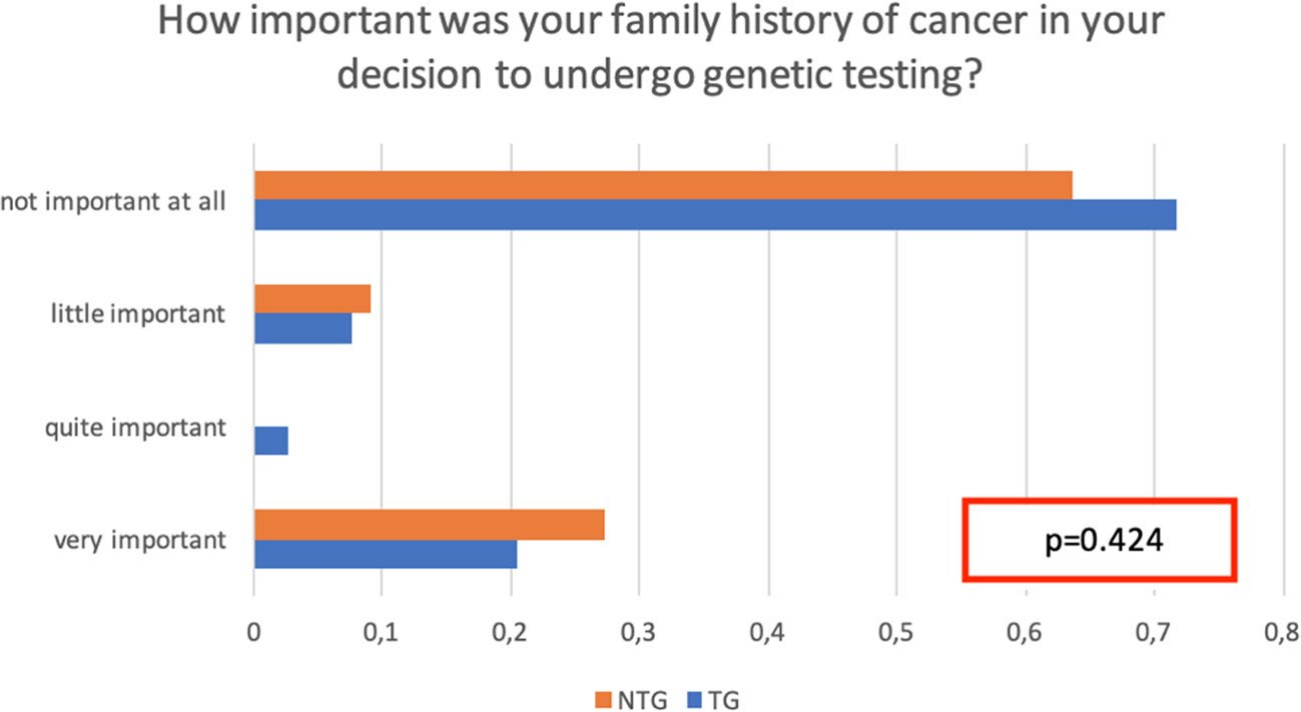
Role of family history of cancer in the decision to undergo genetic testing

During the pre-test counseling, the study participants were asked to rate on a scale from 1 = “very important” to 4 = “it is not important at all,” the level of involvement concerning each proposed question. The role of personal and family history of cancer was investigated, and no differences emerged between the groups in the choice to undergo testing; 20.5% of TG vs 27.3% of NTG patients consider oncological family history to be “very important” (Fig. 1). Personal history of cancer is a decisive factor in the counseling process according to both groups, for 98% of the patients in the TG and 100% of the NTG (*p* = 0.99).

According to both groups, the need to protect their offspring was "very important" in the decision to undertake genetic testing to an almost superimposable degree (64.1 TG vs 54.5 TG, *p* = 0.99). Moreover, regarding the fear of passing on the possible genetic mutation to their offspring, the two groups overlapped, with 33.3% in the TG and 27.3% in the NTG (*p* = 0.7344).

Subjects in the TG were more likely to discuss the possibility of genetic testing with their family (94.9% of the TG versus 54.5% of the NTG, *p* = 0.004). In contrast, 45.5% of NTG considered it inappropriate to inform or discuss pre-test counseling with relatives (*p* = 0.004). The 82.1% of the TG appears to have a family more supportive of genetic testing, compared with 54.5% of the NTG (*p* = 0.105). Only 33.3% of the TG claimed to have been influenced by family members in their decision to take the BRCA1/2 genetic test, in contrast, 54.5% of those who refused the test reported having been influenced by their family (*p* = 0.293), although not statistically significant, these differences appear remarkable.

To assess the impact of the decision to undergo oncogenetic counseling as a distressing experience, the IES was proposed. The results were analyzed using the mean of the answers; higher results correspond to higher levels of distress of the subject. Comparing the mean scores of the two groups, a mean of 19.85 (S.D. 1.11) for the TG, and a mean of 23.36 (S.D. 2.56) for the NTG (*p* = 0.164) has been calculated.

**Fig. 2:**
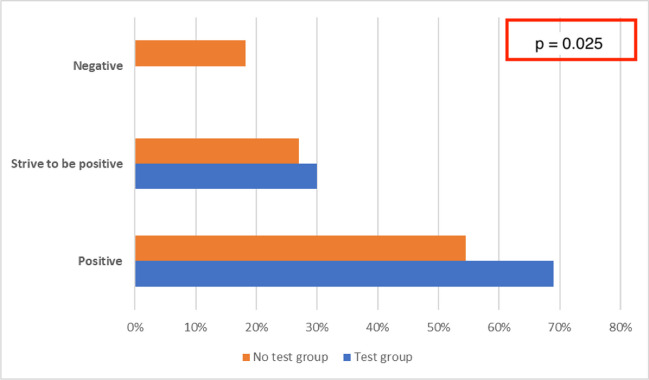
Everyday life attitude of patients under study

Participants in the study were asked to describe their everyday life attitude, thus defining a personal tendency towards positivity or negativity as seen in Fig. 2.

Those who considered themselves positive in dealing with everyday life events were 69.2% of the TG vs 54.5% of the subjects in the NTG. Only in the NTG did 18.2% of subjects claim to have a negative approach to everyday life events. (p = 0.025).

- *Questionnaire C*

Between the NTG, 75% of the subjects felt a sense of fear about the consequences of any information related to genetic testing, both personally and concerning their family, fear of having passed the mutation on to offspring emerged in 37.5% of patients. Sixty-three percent did not think it was fair to involve family members in this decision. More than half of the patients (62.5%) raised logistical issues as a reason for refusing the test: the hospital is far away, and the possibility of potentially carrying a mutation would have meant numerous follow-up visits in case of a positive genetic test result.

## Discussion

In this study, we analyzed the attitudes of subjects with a history of breast and/or ovarian cancer toward the decision of whether to undergo BRCA 1/2 genetic testing.

As in a study by Meijers-Heijboer et al. (Meijers-Heijboer et al. 2000), on 682 unaffected individuals with a high risk for carrying a BRCA mutation, in our sample emerged that adherence to genetic testing is influenced by the age of the patient. In our study, those who agree to undergo genetic testing are younger both at diagnosis and at the time of counseling; although these results are not significant, they show a strong trend. The mean time from cancer diagnosis to oncogenetic counseling was over 10 years for both groups, making the study population extremely selected compared with those who are offered the test at the time of cancer diagnosis. Since the patients under study are long-time survivors, they undertake a different decision-making pathway from those who are prescribed BRCA1/2 genetic testing at the time of cancer diagnosis, for whom the test may also have predictive significance. This may have led some patients to refuse the test, partly out of a desire not to relieve psychological stress at least partially overcome in the past.

Several studies have investigated the predictive role of socio-demographic factors in the uptake of genetic testing in patients with or without a previous personal cancer history. In a study on 60 women of Ashkenazi Jewish background who underwent genetic testing for founder mutations, neither age, educational level, perceived risk, objective risk, nor depression was associated with electing to learn one’s testing result (Andrews et al. 2004). In our sample, education level, occupational status, the presence of comorbidities, including personal history of depression, and use of chronic therapies, alcohol consumption did not play a significant role in the decision to undergo BRCA testing. Likewise, a family history of breast and/or ovarian cancer did not contribute significantly to the decision to undertake testing. In contrast, in another study on 279 members of families with BRCA1-linked hereditary breast-ovarian cancer, testing appears correlated with a greater number of affected first-degree relatives (Lerman et al. 1996).

Adherence to screening campaigns for breast and/or cervical cancer tended to be higher in TG patients, although this result did not reach significance; however, this trend can be explained probably because of a greater risk and prevention awareness of the individuals who are more willing to be tested (71.8% in the TG vs. 45.5% NTG). The greater compliance of patients adhering to screening programs had already been highlighted by He W. et al. (He et al. 2019) in a study regarding adherence to adjuvant hormone therapy in mammography screening participants compared with non-participants. In this study, breast cancer patients not participating in mammography screening were more likely to discontinue adjuvant hormone therapy and to have worse disease-free survival.

The implication of the offspring in the decision to undergo genetic testing has been evaluated in several studies. The psychological and decision-making impact of having children on the decision to test has been highlighted (O’Neill et al. 2015), and many women report seeking BRCA1/2 testing for the benefit of their family members (Tercyak et al. 2002).

In our study, it was observed that the decision to undergo genetic testing might be influenced by having daughters, but not by having male sons, this difference did not appear frankly significant, but a strong trend emerged. This result is to be weighed from the perspective that our patient population has a mean age of 61 years, thus rarely having minor-aged offspring. The patient’s perspective in our study is predominantly from the aspect of mutation transmission and less on the concerns that parents of young children might have in harboring the mutation. Probably then in this perspective, BRCA mutation carrier status may be perceived by patients as more impactful for a daughter than for a son. The population in our study may not know or consider that male offspring with a pathogenic BRCA mutation are themselves at increased risk for breast and prostate cancer. In addition, male offspring with a mutation can pass the mutation on to their daughters, who in turn are at high risk for breast and ovarian cancer.

Disclosure of genetic information about cancer susceptibility has numerous implications for patients but also family members (Lerman et al. 1998; Lehmann et al. 2000), and family context is very important in the choice to pursue an oncogenic pathway (Shkedi-Rafid et al. 2012). TG subjects were more likely to discuss the possibility of genetic testing with their family, while nearly 50% of NTG considered it inappropriate to inform or discuss pre-test counseling with their relatives. The majority of the TG appears to have a family more supportive of genetic testing, but relatively, a small percentage affirmed to have been influenced by the family in their decision to undergo BRCA1/2 testing, thus showing a good level of family cohesion despite declaring greater independence from family influence. In contrast, barely half of the NTG claims to have a family supportive of genetic testing, and most importantly a higher percentage than the TG said they had been influenced by family members in their decision to undergo the BRCA1/2 genetic test. It appears that family cohesion and proper communication with the family can be determining factors in the decision to undergo genetic testing for BRCA mutations. Consequently, it would be advisable to promote proper communication with close family members and also ensure proper information about the family, which is involved together with the consultee, in terms of both the possibility of inheritance of the mutation and the decision-making process. It would therefore be important to promote the presence of one or more family members besides the partner at the time of oncology counseling, possibly including a first- or second-degree relative. As it turned out in our population, the presence of a stable partner seems to discourage the decision to know about their mutational status. In this population, one hypothesis is that patients have dealt with breast or ovarian oncology diagnosis and treatment more than 10 years earlier, the majority being at the time of counseling cured. This may have led them to avoid pursuing the pathway of diagnosis of genetic oncological predisposition to avoid partner involvement in a psychologically and logistically complex new process. Nevertheless, the partner would always be secondarily involved; conversely, first- and second-degree relatives might be personally involved, as they may be carriers of any genetic mutation present in the family. These findings agree with what has been observed in studies of women in which the reason for not disclosing decisions about BRCA testing and the oncogene counseling pathway to family members is a high sense of distress (Segal et al. 2004). Correct information about the risks and benefits of the test should therefore still be extended to both the partner and close family members. The patient's own family and psychological context at the time he or she decides to start oncogenetic counseling are decisive in the choice of whether or not to take the test and also contribute to the establishment of behaviors and psychological status following the receipt of the result (Meiser 2005).

IES scores highlighted that NTG subjects tended to perceive BRCA1/2 genetic testing as a more distressing experience than TG subjects, with a difference that seems noteworthy, although not significant; moreover, NTG subjects show themselves relatively less positive in coping with everyday life than TG subjects. This may highlight how proper management of anxiety and stress in general, and related specifically to genetic testing is a favorable factor. Therefore, the oncogenetic counseling process should be complemented by the possibility of psychological counseling when needed.

Some possible limitations of the study are the small sample size and the fact that the sample adherence rate to genetic testing was high, so a disproportion of the two groups studied (TG and NTG) emerged. Due to the small sample size, several results show a strong trend but do not reach statistical significance. However, the results are supported by existing data from other studies. In fact, some of these trends seemed interesting and useful to provide insights aimed at better understanding our population and the motivations related to the approach to oncogenetic counseling and testing for BRCA1/2 mutations. These trends, together with the frankly significant findings, could indeed be a cue for daily clinical management and an incentive for planning further studies on this topic with a larger population.

## Conclusions

The results of our study show that BRCA1/2 genetic testing is well accepted in a group of long-surviving patients with a previous diagnosis of breast and ovarian cancer, even more than 10 years after the first cancer diagnosis with 78% adherence.

We observed that family cohesion and communication with relatives improve test adherence. Therefore, correct information about the risks and benefits of the test should be extended to both the partner and close family members. The presence of family members, besides the partner, at pre-test counseling should be encouraged; since they are involved in the knowledge of the mutational status of the consultee, this could facilitate decision-making, improve communication and family cohesion, and reduce negative psychological effects.

In conclusion, it would be of interest to conduct oncogenetic counseling campaigns like the one described in this study to expand testing to larger populations, starting with those at higher risk. With the perspective of further broadening the access criteria to genetic testing, it is important to understand how to best approach pre-test counseling even in patients with remote cancer histories. Our study allowed us to screen families that would not otherwise have been identified, because of the long latency from the previous cancer diagnosis. Further studies are needed in this population, with a larger case series and also in light of the observations from this study, evaluating the effect of systemic involvement of family members and psychological counseling.

### Supplementary Information

Below is the link to the electronic supplementary material.

Supplementary file1 (PDF 255 KB)

Supplementary file2 (PDF 42 KB)

Supplementary file3 (DOCX 22 KB)

Supplementary file4 (PDF 56 KB)

Supplementary file5 (PDF 235 KB)

Supplementary file6 (PDF 235 KB)

Supplementary file7 (PDF 155 KB)

Supplementary file8 (PDF 44 KB)

## Data Availability

The data presented in this study are available on request from the corresponding author.
