# Supplementary material for: Factors associated with adherence to BRCA1/2 mutation testing after oncogenetic counseling in long-surviving patients with a previous diagnosis of breast or ovarian cancer
Source: J Community Genet. 2023 Sep 19;14(6):649–56. doi: 10.1007/s12687-023-00671-x (PMC10725406; doi:10.1007/s12687-023-00671-x)
Supplement: Supplementary file 1 — Supplementary file1 (PDF 255 KB) [file 12687_2023_671_MOESM1_ESM.pdf]

Questionnaire A - QUESTIONARIO ANAMNESTICO

1) Età _______

2) Sesso
   ☐ Uomo ☐ Donna

3) Che tipo di tumore ha/ ha avuto in passato?
   ☐ Carcinoma mammario ☐ Carcinoma ovarico ☐ Entrambi

4) A che età le è stato diagnosticato il tumore? ________

5) Ha figli?
   ☐ SI ☐ NO, passi alla domanda 8

6) Se si, di che genere ed età?
   1° figlio ☐M ☐F età _____
   2° figlio ☐M ☐F età _____
   3° figlio ☐M ☐F età _____
   4° figlio ☐M ☐F età _____

7) Intende (o le piacerebbe) averne altri?
   ☐ SI ☐ NO, passi alla domanda 9

8) Intende (o le piacerebbe) averne?
   ☐ SI ☐ NO, passi alla domanda 9

9) Nella sua famiglia ci sono/ci sono stati casi di tumore?
   ☐ SI ☐ NO

10) Se si, indicare in quali soggetti e a che età
   ☐ Genitori ______________
   ☐ Fratelli/sorelle ____________
   ☐ Zie, zii __________

11) Fuma o ha una storia di utilizzo del tabacco?
   ☐ SI ☐ NO

12) Quanto spesso consumi alcolici?
   ☐ Mai ☐ Occasionalmente ☐ Una volta a settimana ☐ Più volte a settimana ☐ Quotidianamente

13) Stato occupazionale:
   ☐ Dipendente ☐ Libero professionista ☐ Casalingo/a ☐ Pensionato/a ☐ Altro

14) Stato civile _____________

15) Anni di scolarizzazione:
☐ Licenza elementare ☐ Licenza media ☐ Licenza superiore ☐ Laurea triennale
☐ Laurea magistrale ☐ Master, Dottorato, etc

16) Ha patologie in atto o pregresse? (Se si, specificare quali) _______________________________

17) Sta attualmente assumendo dei farmaci? (Se si, specificare quali) ___________________________

18) Ha sofferto in passato o soffre attualmente di depressione?
☐ SI ☐ NO

19) Ha completato il ciclo vaccinale contro il Sars-COV2?

☐ No, non mi sono vaccinato/a ☐ Si, ho completato il ciclo vaccinale (con 3° dose effettuata)

☐ Si, ma non ho completato il ciclo vaccinale (2° o 3° dose), perché ____________________________

20) Come valuteresti il tuo stato di salute?

Pessimo<br>1	2	3	4	Ottimo<br>5
○	○	○	○	○

21) Quante ore dorme?
☐ Meno di 5 ore ☐ 5-6 ore ☐ 7-8 ore ☐ 9-10 ore ☐ più di 10 ore

22) Si ritiene una persona positiva nella vita di tutti i giorni?
☐ Si, sono molto positiva/o ☐ No, ma mi sforzo di essere una persona positiva
☐ No, sono una persona negativa

23) Ha aderito di recente al programma di screening "Prevenzione Serena"?
☐ SI ☐ NO, perché ____

24) Se si, quali esami ha svolto? (segnare tutti i test eseguiti, se sono più di uno)
☐ Mammografia bilaterale ☐ Pap-test ☐ Sigmoidoscopia flessibile
☐ Ricerca del sangue occulto nelle feci

25) Sta ancora svolgendo i regolari controlli per il suo tumore?
☐ SI ☐ NO, perché ____

26) Sta effettuando una terapia per il suo tumore?
☐ SI ☐ NO, perché ____

27) Se si, come valuterebbe la sua aderenza alla terapia?

Pessimo<br>1	2	3	4	Ottimo<br>5
◯	◯	◯	◯	◯

28) Prima di essere contattata da noi, aveva già sentito parlare del test genetico per il rischio di cancro alla mammella e/o ovaio?

☐ SI, ero già ben informata sull'argomento ☐ Ne ho sentito parlare, ma non ero ben informata

☐ NO, non ne avevo mai sentito parlare
