# Supplementary material for: Factors associated with adherence to BRCA1/2 mutation testing after oncogenetic counseling in long-surviving patients with a previous diagnosis of breast or ovarian cancer
Source: J Community Genet. 2023 Sep 19;14(6):649–56. doi: 10.1007/s12687-023-00671-x (PMC10725406; doi:10.1007/s12687-023-00671-x)
Supplement: Supplementary file 2 — Supplementary file2 (PDF 42 KB) [file 12687_2023_671_MOESM2_ESM.pdf]

Questionnaire A - ANAMNESTIC QUESTIONNAIRE

1) Age ______

2) Gender

☐ Man ☐ Woman

3) What type of cancer do you have/have you had in the past?

☐ Breast cancer ☐ Ovarian cancer ☐ Both

4) At what age was the tumor diagnosed? ________

5) Do you have children?

☐ YES ☐ NO, go to question 8

6) If yes, what gender and age?

1st child ☐M ☐F age _____

2nd child ☐M ☐F age _____

3rd child ☐M ☐F age _____

4th child ☐M ☐F age _____

7) Do you intend (or would you like) to have more?

☐ YES ☐ NO, go to question 9

8) Do you intend (or would you like) to have any?

☐ YES ☐ NO, go to question 9

9) Is there/were there any cases of cancer in your family?

☐ YES ☐ NO

10) If yes, indicate in which subjects and at what age

☐ Parents _______________

☐ Brothers/sisters _____________

☐ Aunts/uncles ___________

11) Do you smoke or have a history of using tobacco?

☐ YES ☐ NO

12) How often do you drink alcohol?

☐ Never ☐ Occasionally ☐ Once a week ☐ Several times a week

☐ Daily

13) Employment status:

☐ Employee ☐ Freelancer ☐ Housewife ☐ Retired ☐ Other

14) Marital status ______________

15) Years of schooling:

☐ Elementary school certificate ☐ Middle school degree ☐ High school degree

☐ Three-year degree ☐ Master's degree, Doctorate, etc

16) Do you have current or previous conditions?(if yes, specify which)
____

17) Are you currently taking any medications? (If yes specify)
____

18) Have you suffered in the past or do you currently suffer from depression?
☐ YES ☐ NO

19) Have you completed the vaccination cycle against Sars-COV2?
☐ No, I have not been vaccinated ☐ Yes, I have completed the vaccination cycle (with the 3rd dose done) ☐ Yes, but I have not completed the vaccination cycle (2nd or 3rd dose), because _____________________________

20) How would you rate your health?

Very bad				Very good
1	2	3	4	5

21) How many hours do you sleep?
☐ Less than 5 hours ☐ 5-6 hours ☐ 7-8 hours ☐ 9-10 hours ☐ More than 10 hours

22) Do you consider yourself a positive person in everyday life?
☐ Yes, I'm very positive ☐ No, but I try to be a positive person
☐ No, I'm a negative person

23) Have you recently joined the "Prevenzione Serena" screening programme?
☐ YES ☐ NO, because _______________________________________

24) If yes, what exams did you take? (mark all the tests performed, if there are more than one)
☐ Bilateral mammography ☐ Pap smear ☐ Flexible sigmoidoscopy
☐ Search for occult blood in the stool

25) Are you still having your regular checkups for your cancer?
☐ YES ☐ NO, because ____________________________________

26) Are you undergoing therapy for your tumour?
☐ YES ☐ NO, because _____________________________________

27) If yes, how would you evaluate your adherence to the therapy?

Very bad				Very good
1	2	3	4	5

28) Before being contacted by us, had you already heard of the genetic test for the risk of breast and/or ovarian cancer?
☐ YES, I was already well informed on the subject.
☐ I have heard about it, but I was not well informed
☐ NO, I've never heard of it
