# Supplementary material for: Factors associated with adherence to BRCA1/2 mutation testing after oncogenetic counseling in long-surviving patients with a previous diagnosis of breast or ovarian cancer
Source: J Community Genet. 2023 Sep 19;14(6):649–56. doi: 10.1007/s12687-023-00671-x (PMC10725406; doi:10.1007/s12687-023-00671-x)
Supplement: Supplementary file 3 — Supplementary file3 (DOCX 22 KB) [file 12687_2023_671_MOESM3_ESM.docx]

Supplementary file – answers to QUESTIONNAIRE B

**Part 1:** *The context of the decision*

|  |  | **Test-group n.39(%)** | **No-test group n.11(%)** | **p-value** |
| --- | --- | --- | --- | --- |
| - How much your personal history of cancer was decisive in choosing to undergo genetic counseling? | Very important | 26 (66.7) | 8 (72.7) | 0.861 |
|  | Quite important | 10 (25.6) | 2 (18.2) |  |
|  | Little important | 2 (5.1) | 1 (9.1) |  |
|  | Not important at all | 1 (2.6) | 0 (0) |  |
| - If you have cases of cancer in your family, how much did they influence your decision to undergo the genetic counseling? | Very important | 8 (20.5) | 3 (27.3) | 0.424 |
|  | Quite important | 1 (2.6) | 0 (0) |  |
|  | Little important | 3(7.7) | 1 (9.1) |  |
|  | Not important at all | 28 (71.8) | 7 (63.6) |  |
| - How much was the decision to undergo counseling conditioned by the need to protect your children? | Very important | 25 (64.1) | 6 (54.5) | 0.999 |
|  | Quite important | 6 (15.4) | 1 (9.1) |  |
|  | Little important | 0 (0) | 0 (0) |  |
|  | Not important at all | 8 (20.5) | 4 (36.4) |  |
| - How concerned would you be about passing her genetic mutation on to your children? | Very important | 13 (33.3) | 3 (27.3) | 0.7344 |
|  | Quite important | 5 (12.8) | 1 (9.1) |  |
|  | Little important | 6 (15.4) | 2 (18.2) |  |
|  | Not important at all | 15 (38.5) | 5 (45.4) |  |
| - During the phone call you were informed about the increased risk of developing cancer if the genetic test is positive. How decisive was this information in deciding to come to counseling? | Very important | 13 (33.3) | 3 (27.3) | 0.951 |
|  | Quite important | 5 (12.8) | 1 (9.1) |  |
|  | Little important | 6 (15.4) | 2 (18.2) |  |
|  | Not important at all | 15 (38.5) | 5 (45.5) |  |
| - Have you told your family/friends about it? | Yes | 37 (94.9) | 6 (54.5) | **0.004** |
|  | No | 2 (5.1) | 5 (45.5) |  |
| - Is your family in favor of genetic testing for BRCA genes? | Yes | 32 (82.1) | 6 (55.5) | 0.293 |
|  | No | 7 (17.9) | 5 (45.5) |  |
| - How much did your family influence your choice? | Vey | 9 (23.1) | 2 (18.2) | 0.606 |
|  | Quite | 5 (12.8) | 3 (27.3) |  |
|  | Little | 8 (20.5) | 1 (9.1) |  |
|  | Not at all | 17 (43.6) | 5 (45.5) |  |
| - Do you think that the genetic test could be a source of stress in your life? | Vey | 4 (10.3) | 3 (27.3) | 0.269 |
|  | Quite | 6 (15.4) | 1 (9.1) |  |
|  | Little | 8 (20.5) | 2 (18.2) |  |
|  | Not at all | 21 (53.8) | 5 (45.5) |  |
| - Would undergoing testing make you feel calmer and more relieved? | Vey | 17 (43.6) | 1 (9.1) | 0.144 |
|  | Quite | 17 (43.6) | 6 (63.6) |  |
|  | Little | 4 (27.3) | 3 (27.3) |  |
|  | Not at all | 1 (9.1) | 1 (9.1) |  |
| - Would the decision of undergoing the genetic test motivated by the need to quantify your risk of developing cancer? | Vey | 4 (10.3) | 0 (0) | 0.477 |
|  | Quite | 9 (23.1) | 4 (36.4) |  |
|  | Little | 15 (38.5) | 3 (27.3) |  |
|  | Not at all | 11 (28.2) | 4 (36.4) |  |
| - Would you feel psychologically safe in facing the situation of a possible positive test? | Vey | 11 (28.2) | 4 (36.4) | 0.539 |
|  | Quite | 20 (43.6) | 3 (27.3) |  |
|  | Little | 6 (10.2) | 3 (27.3) |  |
|  | Not at all | 2 (5.1) | 1 (9.1) |  |
| - After today's discussion, how much confidence do you have in active surveillance measures? | Vey | 37 (94.8) | 10 (90.9) | 0.625 |
|  | Quite | 2 (5.2) | 1 (9.1) |  |
|  | Little | 0 (0) | 0 (0) |  |
|  | Not at all | 0 (0) | 0 (0) |  |
| - Was the decision to undertake this path of genetic counseling guided by a desire to take care of oneself? | I did it mainly for my health | 3 (7.7) | 1 (9.1) | 0.979 |
|  | I did it for my family | 10 (25.6) | 3 (27.3) |  |
|  | I did it for my health and that of my family | 26 (66.7) | 7 (63.6) |  |

**Part2.** *Impact of event scale.* S.D. Standard Deviation

|  | **Test-group n.39(S.D.)** | **No-test group n.11 (S.D.)** | **p-value** |
| --- | --- | --- | --- |
| Mean values for impact of event scale | 18.95 (1.11) | 23.36 (2.56) | 0.164 |
