# Supplementary material for: Factors associated with adherence to BRCA1/2 mutation testing after oncogenetic counseling in long-surviving patients with a previous diagnosis of breast or ovarian cancer
Source: J Community Genet. 2023 Sep 19;14(6):649–56. doi: 10.1007/s12687-023-00671-x (PMC10725406; doi:10.1007/s12687-023-00671-x)
Supplement: Supplementary file 4 — Supplementary file4 (PDF 56 KB) [file 12687_2023_671_MOESM4_ESM.pdf]

QUESTIONNAIRE B

Part 1: *The context of the decision*

- How much your personal history of cancer was decisive in choosing to undergo genetic counseling?
  ☐ Very ☐ Quite ☐ Little ☐ Not at all

- If you have cases of cancer in your family, how much did they influence your decision to undergo the genetic counseling?
  ☐ Very ☐ Quite ☐ Little ☐ Not at all

- How much was the decision to undergo counseling conditioned by the need to protect your children?
  ☐ Very ☐ Quite ☐ Little ☐ Not at all

- How concerned would you be about passing her genetic mutation on to your children?
  ☐ Very ☐ Quite ☐ Little ☐ Not at all

- During the phone call you were informed about the increased risk of developing cancer if the genetic test is positive. How decisive was this information in deciding to come to counseling?
  ☐ Very ☐ Quite ☐ Little ☐ Not at all

- Have you told your family/friends about it?
  ☐ YES ☐ NO

- Is your family in favor of genetic testing for BRCA genes?
  ☐ YES ☐ NO

- How much did your family influence your choice?
  ☐ Very ☐ Quite ☐ Little ☐ Not at all

- Do you think that the genetic test could be a source of stress in your life?
  ☐ Very ☐ Quite ☐ Little ☐ Not at all

- Would undergoing testing make you feel calmer and more relieved?
  ☐ Very ☐ Quite ☐ Little ☐ Not at all

- Would the decision of undergoing the genetic test motivated by the need to quantify your risk of developing cancer?
  ☐ Very ☐ Quite ☐ Little ☐ Not at all

- Would you feel psychologically safe in facing the situation of a possible positive test?
  ☐ Very ☐ Quite ☐ Little ☐ Not at all

- After today's discussion, how much confidence do you have in active surveillance measures?
  ☐ Very ☐ Quite ☐ Little ☐ Not at all

- Was the decision to undertake this path of genetic counseling guided by a desire to take care of oneself?

☐ Yes, I did it mainly for my health

☐ NO, I did it for my family I did it for my health and that of my family

☐ No, I did because____

IMPACT OF EVENT SCALE

The following is a list of difficulties people sometimes have following stressful life events. Please read each sentence and indicate how much each of the difficulties in question involved you in relation to the telephone consultation and the decision to carry out the genetic test or not.
How affected were these difficulties?

1 Not at all 2 A little bit 3 Moderately 4 Quite a bit 5 Extremely

1. I thought about it when I didn't mean to
2. I avoided letting myself get upset when I thought about it or was reminded of it
3. I tried to remove it from my memory
4. I had trouble falling or staying asleep because of images or thoughts regarding the disease.
5. I had waves of strong feelings about it
6. I had dreams about it
7. I stayed away from reminders about it
8. I felt as if it hadn't happened or wasn't real
9. I tried not to think about it
10. Pictures about the disease popped into my mind
11. Other things kept making me think about it
12. I was aware that I still had a lot of feelings about it, but I didn't deal with them
13. I tried not to think about it
14. Any reminder brought back feelings about it
15. My feelings about it were kind of numb
