# Supplementary material for: Factors associated with adherence to BRCA1/2 mutation testing after oncogenetic counseling in long-surviving patients with a previous diagnosis of breast or ovarian cancer
Source: J Community Genet. 2023 Sep 19;14(6):649–56. doi: 10.1007/s12687-023-00671-x (PMC10725406; doi:10.1007/s12687-023-00671-x)
Supplement: Supplementary file 5 — Supplementary file5 (PDF 235 KB) [file 12687_2023_671_MOESM5_ESM.pdf]

Appendice 3 - QUESTIONARIO B

Parte 1: *Il contesto della decisione*

- Quanto la sua storia personale di tumore è stata determinante nella scelta del test genetico?

☐ Molto
☐ Abbastanza
☐ Poco
☐ Per niente

- Se presenti casi di tumore nella sua famiglia, quanto hanno influito nella scelta di fare il test genetico?

☐ Molto
☐ Abbastanza
☐ Poco
☐ Per niente

- Quanto la scelta di fare il test è stata condizionata dal bisogno di proteggere i propri figli?

☐ Molto
☐ Abbastanza
☐ Poco
☐ Per niente

- In che misura sarebbe preoccupata di trasmettere la sua mutazione genetica ai suoi figli?

☐ Molto
☐ Abbastanza
☐ Poco
☐ Per niente

- Durante la telefonata è stata informata sul rischio aumentato di sviluppare tumore, qualora il test genetico risulti positivo. Quanto quest'informazione è stata determinante nel decidere di venire in consulenza?

☐ Molto
☐ Abbastanza
☐ Poco
☐ Per niente

- Ha provato a parlarne con la sua famiglia/ con i suoi amici?

☐ SI ☐ NO

- La sua famiglia è favorevole al test genetico per la ricerca dei geni BRCA?

☐ SI ☐ NO

- Quanto la sua famiglia ha influenzato la sua scelta?

☐ Molto
☐ Abbastanza
☐ Poco
☐ Per niente

- Ritiene che il test genetico possa essere una fonte stress nella sua vita?
  ☐ Molto
  ☐ Abbastanza
  ☐ Poco
  ☐ Per niente

- Eseguire il test la farebbe sentire più tranquilla e sollevata?
  ☐ Molto
  ☐ Abbastanza
  ☐ Poco
  ☐ Per niente

- La scelta di eseguire il test genetico è stata mossa dal bisogno di *quantificare* il suo rischio di sviluppare tumore?
  ☐ Molto
  ☐ Abbastanza
  ☐ Poco
  ☐ Per niente

- Si sente sicura psicologicamente nell'affrontare la situazione di un eventuale test *positivo*?
  ☐ Molto
  ☐ Abbastanza
  ☐ Poco
  ☐ Per niente

- Dopo il colloquio odierno, quanta fiducia ha nelle misure di sorveglianza attiva?
  ☐ Molta
  ☐ Abbastanza
  ☐ Poca
  ☐ Per niente

- La scelta di intraprendere questo percorso di consulenza genetica è stato guidato da un desiderio di prendersi cura di se?
  ☐ Si, l'ho fatto principalmente per la mia salute ☐ NO, l'ho fatto per la mia famiglia
  ☐ L'ho fatto per la mia salute e per quella della mia famiglia
  ☐ No, l'ho fatto perché____

Appendice 5: IMPACT OF EVENT SCALE

La seguente è una lista di difficoltà che le persone hanno talvolta in seguito ad eventi stressanti della vita. Legga per favore ogni frase ed indichi quanto l'ha coinvolta ognuna delle difficoltà in questione relativamente al consulto telefonico e alla decisione di eseguire o meno il test genetico.

Quanto è stato coinvolto da queste difficoltà?

① = per niente ② = un poco ③ = moderatamente ④ = abbastanza ⑤ = tanto

1. Ci ho pensato senza averne l'intenzione.

   ① ② ③ ④ ⑤

2. Ho evitato di arrabbiarmi quando ci ho pensato o quando mi è stato ricordato.

   ① ② ③ ④ ⑤

3. Ho provato a rimuoverlo dalla memoria.

   ① ② ③ ④ ⑤

4. Ho avuto difficoltà ad addormentarmi o a rimanere addormentato, a causa di immagini o pensieri riguardanti la malattia.

   ① ② ③ ④ ⑤

5. Ho avuto ondate di forti sentimenti a riguardo.

   ① ② ③ ④ ⑤

6. Ho fatto sogni a riguardo.

   ① ② ③ ④ ⑤

7. Sono rimasto lontano da cose che potevano ricordarmelo.

   ① ② ③ ④ ⑤

8. Mi sentivo come se non fosse successo o non fosse reale.

   ① ② ③ ④ ⑤

9. Ho cercato di non parlarne.

   ① ② ③ ④ ⑤

10. Immagini della malattia entravano nella mia mente all'improvviso.

    ① ② ③ ④ ⑤

11. Altre cose hanno continuato a farmici pensare.

    ① ② ③ ④ ⑤

12. Ero consapevole del fatto che avevo ancora molti sentimenti a riguardo, ma non li ho affrontati.

    ① ② ③ ④ ⑤

13. Ho cercato di non pensarci.

① ② ③ ④ ⑤

14. Qualsiasi promemoria mi procurava sentimenti a riguardo.

① ② ③ ④ ⑤

15. Provo apatia quando ci penso.

① ② ③ ④ ⑤
