# Supplementary material for: Factors associated with adherence to BRCA1/2 mutation testing after oncogenetic counseling in long-surviving patients with a previous diagnosis of breast or ovarian cancer
Source: J Community Genet. 2023 Sep 19;14(6):649–56. doi: 10.1007/s12687-023-00671-x (PMC10725406; doi:10.1007/s12687-023-00671-x)
Supplement: Supplementary file 7 — Supplementary file7 (PDF 155 KB) [file 12687_2023_671_MOESM7_ESM.pdf]

Appendice 4 - QUESTIONARIO C

Parte 1: *Il contesto della decisione*

- Quanto la sua storia personale di tumore è stata determinante nella scelta del test genetico?

☐ Molto
☐ Abbastanza
☐ Poco
☐ Per niente

- Se presenti casi di tumore nella sua famiglia, quanto hanno potuto influire nella scelta di fare il test?

☐ Molto
☐ Abbastanza
☐ Poco
☐ Per niente

- Durante la telefonata è stata informata sul rischio aumentato di sviluppare tumore, qualora il test genetico risulti positivo. Quanto quest'informazione è stata determinante nel decidere di venire in consulenza?

☐ Molto
☐ Abbastanza
☐ Poco
☐ Per niente

- Ha provato a parlarne con la sua famiglia/ con i suoi amici?

☐ SI ☐ NO

- La sua famiglia è favorevole al test genetico per la ricerca dei geni BRCA?

☐ SI ☐ NO

- Quanto la sua famiglia ha influenzato la sua scelta?

☐ Molto
☐ Abbastanza
☐ Poco
☐ Per niente

- Quali tra queste motivazioni l'hanno spinta a rifiutare il test? (può selezionare più risposte)

☐ Lo scopo del test genetico non mi è chiaro

☐ Non ho voglia di fare il test genetico

☐ Non mi sento in grado di affrontare lo stress delle visite ospedaliere

☐ Ho paura delle conseguenze di qualsiasi informazione negativa, per me o per la mia famiglia

☐ Non mi sembra giusto coinvolgere la mia famiglia

☐ Mi sento in colpa verso la mia famiglia

☐ Ho paura di avere la mutazione e di averla trasmessa ai miei figli

☐ Non voglio sapere se ho una mutazione genetica che mi predispone al cancro

☐ Il test è una perdita di tempo in quanto non migliorerà la qualità della mia vita

☐ Il test è una perdita di tempo perché ho già il cancro

☐ L'ospedale è troppo lontano

☐ Non ho tempo per fare tutte le visite necessarie previste dal programma di sorveglianza

☐ Fare il test non mi impedirà di ammalarmi di cancro e morire

☐ Non sento di poter affrontare lo stress delle analisi del sangue

☐ Se dovessi essere portatore di una mutazione non potrei sopportare l'idea di sottopormi a frequenti controlli

☐ Se dovessi essere portatore di una mutazione non potrei sopportare l'idea di sottopormi a chirurgia
profilattica

☐ Non posso tollerare l'idea di un'eventuale chirurgia profilattica perché ho paura dell'anestesia

☐ Altro: ____
