# Supplementary material for: Factors associated with adherence to BRCA1/2 mutation testing after oncogenetic counseling in long-surviving patients with a previous diagnosis of breast or ovarian cancer
Source: J Community Genet. 2023 Sep 19;14(6):649–56. doi: 10.1007/s12687-023-00671-x (PMC10725406; doi:10.1007/s12687-023-00671-x)
Supplement: Supplementary file 8 — Supplementary file8 (PDF 44 KB) [file 12687_2023_671_MOESM8_ESM.pdf]

QUESTIONNAIRE C

Part 1: *The context of the decision*

- How much your personal history of cancer was decisive in choosing about genetic testing?
  ☐ Very ☐ Quite ☐ Little ☐ Not at all

- If you have cases of cancer in your family, how much could they influence your decision about genetic testing?
  ☐ Very ☐ Quite ☐ Little ☐ Not at all

- During the phone call she was informed about the increased risk of developing cancer if the genetic test is positive. How decisive was this information in deciding to come to counseling?
  ☐ Very ☐ Quite ☐ Little ☐ Not at all

- Have you tried to tell your family/friends about it?
  ☐ YES ☐ NO

- Is your family in favor of genetic testing for BRCA genes?
  ☐ YES ☐ NO

- How much did your family influence your choice? Very
  ☐ Very ☐ Quite ☐ Little ☐ Not at all

- Which of these reasons prompted you to refuse the test? (can select multiple answers)

☐ The purpose of the genetic test is not clear to me
☐ I don't want to do the genetic test
☐ I feel unable to deal with the stress of hospital visits
☐ I fear the consequences of any negative information, for me or my family. It doesn't seem right to involve my family
☐ I feel guilty towards my family
☐ I am afraid that I have the mutation and have passed it on to my children
☐ I don't want to know if I have a genetic mutation that predisposes me to cancer
☐ The test is a waste of time as it will not improve my quality of life
☐ The test is a waste of time because I already have cancer
☐ The hospital is too far away
☐ I don't have time to make all the necessary visits required by the surveillance program
☐ Getting tested won't stop me from getting cancer and dying
☐ I don't feel I can handle the stress of blood tests
☐ If I were to carry a mutation I could not bear the idea of undergoing frequent checks
☐ If I were to carry a mutation I could not bear the idea of undergoing surgery prophylactic
☐ I cannot tolerate the idea of any prophylactic surgery because I am afraid of anesthesia
☐ Other:
____
